# Norms for a Pictographic System: The Aragonese Portal of Augmentative/Alternative Communication (ARASAAC) System

**DOI:** 10.3389/fpsyg.2018.02538

**Published:** 2018-12-14

**Authors:** Daniela Paolieri, Alejandra Marful

**Affiliations:** ^1^Mind, Brain and Behavior Research Center, University of Granada, Granada, Spain; ^2^Department of Psychology, University of Jaén, Jaén, Spain

**Keywords:** augmentative and alternative communication, pictographic system, picture naming, Spanish normative study, language production

## Abstract

Different systems are used to facilitate communication for people with speech problems. Among these, pictographic systems offer an extraordinary solution for many people with severe communication disorders; for example, people with autism spectrum disorders, aphasia, cognitive impairment, cerebral palsy, etc. The pictographic system called Aragonese Portal of Augmentative and Alternative Communication (ARASAAC http://arasaac.org), freely distributed under the Creative Commons License (BY-NC-SA), is an important reference in many countries. Although these images are widely used, there are no previous studies on their reliability and validity. In order to obtain a useful tool in the clinical context, scores of name agreement, H index, tip-of-the-tongue responses, conceptual familiarity, image agreement, visual complexity, and response times were collected for the 295 most frequent images in the ARASAAC dataset. The psychometric analyses showed adequate validity and reliability values. The regression analysis indicated that naming times were explained by picture-name agreement, age of acquisition, and conceptual familiarity, while the tip-of-the-tongue states were mainly predicted by picture-name agreement and name agreement. In conclusion, these norms from the ARASAAC dataset offer a valuable tool for clinical intervention as well as for psycholinguistic research.

## Introduction

Among all possible ways of responding to the basic need of human communication, pictographic language offers an extraordinary solution for many people with serious expressive or receptive communication disorders (Beukelman and Mirenda, 2013). The pictographic language transmits a message with simplicity and clarity, beyond cultural, linguistic, or cognitive boundaries, using images or symbols that represent people, objects, or ideas (see Romski et al., 2015, for a review on augmentative communication intervention). The Aragonese Portal of Augmentative and Alternative Communication (ARASAAC) pictographic system (Palao, 2013) was implemented in 2007 as part of a project financed and coordinated by the Department of Education, Culture and Sports of the Government of Aragon and coordinated by the General Direction of Innovation, Equity, and Participation with the collaboration of the Aragon Center for Technologies for Education (CATEDU) and the Public School of Special Education in Alborada (Zaragoza), Spain. In recent years, this system has become an important reference in countries such as Spain, France, Brazil, Italy, Finland, Germany, and Belgium, and it is becoming increasingly more widespread worldwide (United States, Senegal, Qatar, etc.) (Romero Corral and Marcos Rodrigo, 2016). The dictionary of Spanish words within the complete set of pictograms has been translated to 17 languages: Arabic, Chinese, Romanian, Russian, Polish, Bulgarian, English, French, Italian, German, Portuguese, Brazilian, Croatian, Euskera, Galician, Catalan, and Valencian. All the pictograms and their corresponding names (in color *N* = 18206; 2018, July) are freely distributed under the Creative-Commons License (BY-NC-SA). This has allowed the presence of ARASAAC in different areas such as education, hospitals, care for the elderly, adaptation of documents, accessibility of a means of communication, accessible tourism, or signage. Proof of this relevance and diffusion are the numerous awards it has received, including candidacy for the Prince of Asturias Awards for Communication and Humanities.

Beyond these merits, the ARASAAC database seems to be one of the most appropriate augmentative alternative communication systems when compared with other pictographic systems. Thus, the research of Cabello and Bertola (2015) was focused on the analysis of iconicity and transparency of 38 ARASAAC pictograms taken from 4 grammatical categories: names, verbs, adjectives, and linguistic symbols. This study compared this set of pictograms with other well-known pictographic systems, such as Picture Communication Symbols (Johnson, 1981) and Bliss (Bliss, 1965; Hehner, 1980). The results showed that the ARASAAC pictograms had a greater degree of transparency and iconicity than the Picture Communication Symbols (with the exception of transparency for linguistic symbols, where there was no difference) and Bliss systems. Consequently, ARASAAC is one of the preferred augmentative alternative communication systems.

The usefulness of this database can, however, be constrained by several limitations that modulate language production. First, the provided names in the ARASAAC dataset have been assigned to a given picture according to subjective criteria, and are not based on controlled and systematic normative studies. Therefore, the corresponding name-picture in this dataset may not be the most appropriate, and consequently, it is possible that professionals might employ a label for a given pictogram that differs from the meaning assigned by the patients. Second, psycholinguistic variables have been proven to affect the development of name production. Some of these variables refer to general properties of a concept (e.g., word frequency, concreteness, word length, age of acquisition etc.) and can be easily obtained from existing databases that contain normative data for wide sets of words, for example EsPal (Duchon et al., 2013), NIM (Guasch et al., 2013) or NIPE (Díez et al., 2014). Other variables, however, are strongly tied to the selected stimuli in a dataset and need to be derived from the specific group of pictures in which we are interested. Thus, for example, the relation between the picture of a concept and our mental image of this concept (image agreement), conceptual familiarity and the visual complexity of the picture (Snodgrass and Vanderwart, 1980) depend on the images we are presented with. These indices, among others, can explain why one picture is easily named and/or recognizable, but a similar one is not.

Most views of word production (e.g., Dell, 1986; Glaser, 1992; Caramazza, 1997; Levelt et al., 1999; Bonin et al., 2002) assume that object naming involves several processing levels to reach the correct articulation and execution of the necessary motor program. According to Levelt et al. (1999) influential model, the first level is the perceptual analysis of the visual input, which results in the activation of structural representation and in the recognition of the object. Next, the activation spreads to the central level of lexical access, with the retrieval of semantic/conceptual information, lexical (“lemma”) selection and lexeme formation, with the composition of the phonological information. Each of the variables for which we recollect the norms (name agreement, visual complexity, image agreement, conceptual familiarity) affects one (or more) of these processing levels, as we specify in the following part, and it is also desirable to know the specific values of these dimensions for the ARASAAC set of pictures.

To achieve this aim, we collected normative data for a set of pictures from the ARASAAC system. For example, two indices for name agreement were calculated: first, the name agreement index refers to the percentage of modal name responses out of the total responses for a given picture. Given that name agreement is not sensitive to the heterogeneity of the names produced for a picture, we also calculated the preferable name agreement index, called the H statistic (Snodgrass and Vanderwart, 1980).

Lower levels of name agreement typically produce slower responses to a picture (Vitkovitch and Tyrrell, 1995; Barry et al., 1997; Bonin et al., 2002; Shao et al., 2014). Two main sources of this effect have been proposed: uncertainty regarding pictures or alternative names of depicted objects. The first one seems to have its locus at the stage of object recognition, whereas the second one, regarding the activation of more than one alternative name, occurs after conceptual access at the name retrieval stage (Vitkovitch and Tyrrell, 1995; Bonin et al., 2002; Cheng et al., 2010).

Also located in the structural level, visual complexity is defined as the number of lines or the intricacy of the lines. The more visually complex a picture is, the harder it is to process (Hartje et al., 1986), although not much experimental evidence has found this factor to be a significant predictor of picture-naming speed (e.g., Barry et al., 1997; Cycowicz et al., 1997; Bonin et al., 2002, 2003; but see Ellis and Morrison, 1998; Alario et al., 2004; and Perret and Bonin, 2018 for a recent meta-analysis). However, we consider it relevant to include this variable in our database, as it can have some effect at the level of picture’ visual characteristic processing, especially considering that the pictures in the ARASAAC dataset can vary according to various physical features (shape, surface details, background, color etc.). Moreover, image agreement refers to the degree of correspondence between the provided picture for a given concept and the mental image we have for this concept in the corresponding stored structural representation (Barry et al., 1997). Pictures with higher values of image agreement produce faster responses in a naming task (Barry et al., 1997; Bonin et al., 2002).

Finally, conceptual familiarity was defined as the degree of physical or mental contact with an object, influencing the facility of access to semantic/conceptual representations. Despite the mixed evidence observed on the impact of this variable on the picture-naming performance of healthy participants (e.g.,Snodgrass and Yuditsky, 1996; Cuetos et al., 1999; but see also Bonin et al., 2002; Alario et al., 2004), the importance of conceptual familiarity in picture naming has been highlighted in a recent meta-analysis (Perret and Bonin, 2018) and in the picture-naming performance of aphasic patients (e.g., Hirsh and Funnell, 1995), with direct relevance for a tool for communication disorders like ARASAAC.

In sum, for a set of the most frequent pictures in the ARASAAC system, the modal name, name agreement index, H index, number of ToT states, image agreement scores, visual complexity scores, conceptual familiarity scores and naming times were collected. We expect that these data were useful in both clinical and research realms.

## Materials and Methods

### Participants

A total of 219 students from the University of Jaén in Spain participated in this experiment in exchange for course credits. All were native Spanish speakers and had normal or corrected vision. There were different participants for each of the main tasks reported below. All participants gave written informed consent. This protocol was approved by the University of Jaén (Spain) ethics committee.

### Materials and Procedure

A set of words from the ARASAAC dataset and their corresponding pictures were included in this normative study. As a general outline, we first selected a set of concepts (word selection phase) and their corresponding pictures (picture selection phase). Later, pictures were presented in isolation in a written naming task (name agreement task) and next to these pictures, the most commonly produced name for each picture was selected for the following phases. Here, conceptual familiarity (conceptual familiarity task), image agreement (image agreement task) and visual complexity (visual complexity task) scores and response times, as well as other related measures (response time task), were collected. Stimulus presentation and data collection were controlled by E-prime software (version 2.0; Schneider et al., 2012).

#### Word Selection Phase

From the color pictogram catalog at the ARASAAC website^1^ we selected the 300 common names and verbs that were rated as more frequent in the ESPAL database (Duchon et al., 2013). Prepositions, conjunctions, adverbs, adjectives, proper names, and words without concrete values in ESPAL were previously eliminated from this set of words. A deeper revision of the resulting set of stimuli ended with the elimination of 1 item, since there was not a pictogram for the general concept (i.e., there were pictograms for the numbers 1, 2, 3, etc., but not for the token “number”) and 4 items with double grammatical functions (name/verb and adjective) where the corresponding pictogram only reflected the adjectival meaning. Consequently, out of the 300 initial stimuli, 295 items passed to the following phase. These stimuli were downloaded between December 2016 and January 2017.

#### Picture Selection Phase

Only one picture was selected for each collected word. Given that in this dataset, there were some cases where words were associated with more than one picture, a set of criteria for selecting a unique picture were followed. First, we chose the picture that corresponded in gender and number to the selected words (e.g., if the word was the masculine “abogado” (lawyer) we eliminated the pictures of a woman lawyer and the pictures of a group of lawyers). Second, if for one word there were pictures that referred to general terms and pictures that referred to cases, we selected the picture of the most general term. For example, the ARASAAC database contained different pictures for the word “die.” Thus, there were (a) one picture with a cube rotted at an angle that showed some of its sides or (b) six pictures of a cube showing one only side with one, two,… or six dots. In order to select an only picture, we considered that the first one was more general and a better representation of the general concept “die” and consequently, we selected this item. Third, we also selected the pictures most appropriate for the geographic context of these norms, thus, for the countries and flags, we selected the items that corresponded to Spain. Fourth, in the cases of pictures that were associated with ambiguous words (i.e., words with more than one meaning or sense; for example, in Spanish, *banco* means bank but also bench), we selected the most frequent meaning based on the databases for ambiguous words from Fraga et al. (2017) and Gómez-Veiga et al. (2010). Out of the 28 words identified as ambiguous, two showed a tie in the frequency of production of their different senses or meanings, and 15 did not appear in either of these norms. This implied that there were no objective criteria to find an individual picture to 17 (15+2) ambiguous words. To select only one picture for these ambiguous words and for the rest of the words where more than one pictured referred to the same item and did not fulfill any of the four previously described criteria, we conducted a preliminary study in which each word and the different associated pictures appeared in a picture selection task. In total, 155 words were selected for this task. Subsequently, a sample of 41 participants (34 females, mean age = 20.98, *SD* = 1.93) were presented with each word beside their corresponding pictures (from 2 to 11 pictures per word). The pictures associated with each word were numbered and appeared centered on the computer screen above the corresponding word. Each slide (preceded by a 1-s fixation point) appeared in random order for each participant for 8 s, or until a response was given. Frequency and response times were recorded. Participants indicated which picture was the most related to the word using the keyboard. For each word, the most frequently selected picture (or the pictures with fastest responses in the case of tied results) was selected to be employed in the following phase. On average, these pictures were selected by the 66.4% of the participants (*SD* = 18.4; range = 26.8–100%). Mean RTs for the responses to these items were 3153 ms (*SD* = 816; range = 1989–5601 ms).

#### Written Name Agreement Task

The previously selected pictures appeared in a written name agreement task. A total of 39 participants (29 females, mean age = 21.23, *SD* = 4.22) were presented with the 295 pictures and, following Snodgrass and Vanderwart (1980) procedure, participants were instructed to write the first name that came to mind (without explicit reference to respond as fast as possible). If participants were not able to respond, (they did not know the picture, they knew the picture but not its name; they knew the picture name, but they suffered a ToT experience^2^) using an apposite option on the computer’s numeric keyboard. The stimuli appeared in a random order for each participant. Each trial started with a fixation point (300 ms), followed by a picture for name generation (6 s), a blank slide (300 ms), and a slide to indicate the reason for a non-response (5 s) when appropriate.

For each picture, the most frequently produced instances were selected to be employed in the following phase. In the few cases where there was a tie between the frequency of two instances (4% of the total), the one associated with faster response times was preferred (but note that we did not instruct participants to respond as quickly as possible).

In two cases, the fastest response was not selected by error; these words are highlighted by an asterisk in the data set.

#### Index Recollection

The previously selected stimuli presented in the index recollection tasks appeared in a random order for each participant, using procedures like the ones employed in other studies (Bonin et al., 2008; Marful et al., 2018).

##### Conceptual familiarity task

Conceptual familiarity ratings were collected by following Barry et al. (1997) and Cuetos et al. (1999) procedure. Thirty participants (26 females, mean age = 22, *SD* = 5.21) were instructed to rate the conceptual familiarity of each picture defined as ‘the degree to which you come in contact with, or think about, the thing depicted’. Thus, after a fixation point (500 ms), the picture appeared for 4 s or until a response, and participants rated its conceptual familiarity on a five-point Likert scale (1 = very unfamiliar, 5 = highly familiar).

##### Image agreement task

In this task, 34 participants (29 females, mean age = 20.35, *SD* = 1.62) indicated the extent to which a presented picture corresponded to the mental image they had of the corresponding concept (e.g., Marful et al., 2015). Thus, after a fixation point (500 ms), the corresponding word appeared on the center of the screen (2 s). After that, a fixation point was showed and participants were instructed to create their mental image of this concept (5 s). Finally, the picture was presented for 4 s or until a response, and participants rated the agreement between their mental image and the presented picture on a 5-point Likert scale (1 = low agreement, 5 = high agreement).

##### Visual complexity task

Following previously established procedures (e.g., Cuetos and Alija, 2003; Bonin et al., 2004), 35 participants (30 females, mean age = 21.65, *SD* = 5.24) were instructed to rate the complexity of each picture. They were told to rate the picture itself rather than the complexity of the concept that it represented. Visual complexity was defined as the number of details of the picture or the intricacy of the lines. Thus, after a fixation point (500 ms), the picture appeared for 4 s or until a response, and participants rated its visual complexity in a 5-point Likert scale (1 = very simple, 5 = very complex).

**Table 1:** Descriptive statistics in the normative study.

	Name agreement	H index	Conceptual familiarity	Image Agreement	Visual Complexity	Phonemes	ToTs	RTs	AoA
Mean	0.5	2.04	3.6	4.14	2.38	6.2	0.89	1097	2.66
Standard Deviation	0.25	0.92	0.8	1.2	1.46	2.03	1.34	236	0.80
Asymmetry Percentile	0.346	−0.345	−0.84	−1.01	0.25	0.69	3.01	0.97	0.88
25	0.29	1.3	3.21	3.89	2	5	0	925	2.16
50	0.46	2.16	3.73	4.24	2.36	6	0	1059	2.60
75	0.72	2.77	4.23	4.53	2.76	7	1.00	1233	2.96
Reliability	0.93	0.91	0.94	0.91	0.88	–	–	0.84	–

**Table 2:** Correlations.

	Name agreement	H	Conceptual familiarity	Image agreement	Visual complexity	Phonemes	AoA	ToTs
H	−0.931^∗∗^							
Conceptual familiarity	0.433^∗∗^	−0.351^∗∗^						
Image agreement	0.486^∗∗^	−0.413^∗∗^	0.462^∗∗^					
Visual complexity	−0.166^∗∗^	0.148^∗^	−0.017	−0.049				
Phonemes	−0.109	0.157^∗∗^	0.152^∗∗^	0.068	0.199^∗∗^			
AoA	−0.138	0.170^∗^	−0.086	−0.110	0.268^∗∗^	0.348^∗∗^		
ToTs	−0.360^∗∗^	0.290^∗∗^	−0.204^∗∗^	−0.349^∗∗^	0.121^∗^	0.006	0.120	
RT	−0.335^∗∗^	0.334^∗∗^	−0.294^∗∗^	−0.415^∗∗^	0.227^∗∗^	0.122^∗^	0.355^∗∗^	0.266^∗∗^

*^∗∗^p < 0.01; ^∗^p < 0.05.*

#### Response Time (RT) Task

The previous 295 pictures were presented to 40 participants (34 females, mean age = 20.86, *SD* = 2.54) in a response time naming task using a vocal key. Participants were instructed to name each picture as soon as possible without error or hesitation, or they had to indicate the reason in case they were not able to produce a response (they did not know the picture, they knew the picture but not its name, or they knew the picture name, but they suffered a ToT experience) using an appropriate choice on the computer’s numeric keyboard. One randomized list was created and divided in two blocks counterbalanced across participants. Each trial started with a fixation point (500 ms), followed by a picture to name (4 s), a slide to indicate the reason for a non-response when appropriate (4 s), and a blank inter-trial slide (2 s). Stimuli appeared with breaks every 20 assays. Naming errors, hesitations, and voice-key failures were recorded by the experimenter.

## Results

This dataset is available in Supplementary Table S1, that contains the modal name of each picture and its English translation, the name of the picture in the ARASAAC norms followed by its English translation, a column indicating if the modal name and the name provided in ARASAAC coincide, the name of the file for each picture, two measures of name agreement (name agreement scores and H scores), means of conceptual familiarity, visual complexity, image agreement, number of ToTs, and response times (RT). The number of phonemes of the modal name and values of age of acquisition (AoA) from the ESPAL database (Duchon et al., 2013) and Davies et al. (2013), respectively, were also provided. Unless otherwise indicated, item analyses were performed averaging or counting over subjects.

### Validity and Reliability

Finally, in the last column, a measure of the validity of the different scales is provided. Since this is the first normative study with ARASAAC pictograms collected in Spain, validity cannot be tested by comparison to an external study. However, given that two different samples of subjects performed name generation tasks when presented the same set of pictures (i.e., participants produced written names in the proper name agreement task and named pictures vocally in the RT task), we conducted a validity test on these data. Although correlational analyses have traditionally been carried out to estimate this psychometric index, when the assignment involves a generation task, other indices seem more convenient (e.g., see Yoon et al., 2004, for a list of possible limitations of correlational analysis in a similar context). Consequently, we calculate the Hellinger affinity (HA) scores for each concept (see the equation below and Lausen et al., 2005, for a more complete description). This index has also been calculated in other normative studies that included generation tasks (e.g., Yoon et al., 2004; Marful et al., 2015).

Hellinger affinity indicates the degrees of overlap between two distributions (the written name agreement and the oral name agreement data in this case), and its value range from 1 (two identical distributions) to 0 (indicating no overlap between the distributions). For each concept, HA is calculated by finding the square root of the product of the two distribution proportions (*p_i_, q_i_)*. Results indicate a high degree of overlap between the two naming tasks (mean = 0.78, *SD* = 0.14); in fact, HA values higher than 0.50 were obtained for 95% of the concepts (see Supplementary Table S1). Taken together, these data confirmed the general validity of the normative study.

$$\text{HA}(p,\;q)=\sum_{i=1}^{\text{n}}\sqrt{p_{\text{i}}q_{\text{i}}}$$

The reliability of the different scales was evaluated employing the split-half method. To this aim, participants were randomly divided into two equal groups in each scale, and the values obtained were correlated employing the Spearman–Brown correction. High levels of reliability were observed in all the scales as depicted in Table 1 (rank 0.84–0.93, average = 0.89).

### Descriptive Statistics

Table 1 contains the means of the descriptive statistics for the different tasks. First, name agreement scores for each picture were calculated.

As described below, two indices of name agreement were computed. First, we calculated the name agreement index, that is, the percentage of modal name responses out of the total responses for a given picture. Given that NA is not sensitive to the heterogeneity of the names produced for a picture, we also calculated the preferable name agreement index, called the H statistic (Snodgrass and Vanderwart, 1980). *H*-values close to 0 indicate a high degree of agreement between participants, while higher values show a reduced degree of agreement. The formula used to calculate H scores is depicted below: *K* refers to the number of different names provided for each picture and *p_i_* is the proportion of responses to each name. The three categories of naming problems (i.e., “do not know the picture,” “do not know its name,” or a “ToT” state), that were included when calculating proper name agreement were eliminated when calculating H.

$$\text{H}=\sum_{\text{i}=1}^{\text{K}}p_{\text{i}}\log_{2}(1/p_{i})$$

Results for these two indices showed a low level of agreement accompanied by a high variability in these values (see Table 1 for descriptive statistics). The name agreement distribution showed a positive asymmetry, illustrating that low levels of name agreement were more frequent than high levels of name agreement. In the same sense, the distribution of H scores showed a negative asymmetry, indicating that high H scores were more frequent. As traditionally observed, there was a significant negative correlation between the name agreement and the H scores (*r* = 0.93, *p <* 0.0001).

We also compared the total number of coincidences between the modal category obtained in the name agreement task and the name provided by the ARASAAC norms. Out of 295, there were 188 cases (63.7%) where these two names differed.

The results of the conceptual familiarity scores indicated that, on average, participants rated the selected pictures with a medium level of familiarity. These values were negatively distributed; that is, higher values of familiarity were more frequent (see Table 1).

Mean values of Image Agreement indicate a high level of correspondence between the picture and the mental image of the concept. The distribution of the values showed a negative asymmetry, indicating that higher values of correspondence were more frequent.

Visual complexity scores showed that the selected pictures were, on average, considered relatively simple (see Table 1). Supporting this idea, lower values of complexity were more frequent in the data distribution as revealed by the negative asymmetry value.

ToTs were rarely elicited by these stimuli (*M* = 0.89) and the distribution showed a positive asymmetry; low numbers of ToTs were more frequent.

### Response Times and ToTs

Similar to the written name agreement task, responses during the RT task were highly variable. We employed the same cut-offs used in Bonin et al. (2008) and in Marful et al. (2018). Thus, we deleted data from participants with less than 21% of correct responses (a total of 3 participants) and RTs that exceeded 2 SD from each item’s mean.

Interestingly, RTs correlated with all indices (see Table 2). Concretely, RT was positively correlated with the H index, visual complexity, number of phonemes, AoA, and ToTs, and negatively with name agreement, conceptual familiarity, and image agreement. The H index correlated positively with visual complexity, number of phonemes, AoA, and ToTs and, negatively with name agreement and conceptual familiarity. Name agreement correlated positively with image agreement and conceptual familiarity and negatively with visual complexity and ToTs. Conceptual familiarity and ToTs were negatively correlated while conceptual familiarity and image agreement correlated positively. Finally, visual complexity correlated positively with number of phonemes, AoA, and ToTs, and number of phonemes correlated positively with conceptual familiarity and AoA.

All the variables with the exception of the H index (that were strongly correlated with name agreement) were submitted as independent variables to a step-wise regression analysis with response times as dependent variable^3^. In the stepwise regression analysis, each independent variable with the smallest *p*-value was entered, and variables already in the model were removed if their probabilities were sufficiently large. This method terminated when no more variables were eligible for inclusion or removal (IBM SPSS, Version 20).

Results indicated that image agreement, AoA and conceptual familiarity predicted RTs; see Table 3. When only exemplars with higher levels of name agreement were selected (i.e., values equal to or higher than 0.50), results indicated that visual complexity, name agreement, image agreement, and AoA explained the variability in response times.

A different step-wise regression analysis was carried out with the number of ToTs as dependent variables and the rest of the variables, with the exception of the H index, as independent variables. Results indicated that the best model included image agreement and name agreement as predictors. When only values equal to or higher than 0.50 in the name agreement scores were selected, image agreement and visual complexity entered in to the model. See Table 4.

**Table 3:** Step-wise regression with RT as a dependent variable.

Variables	*B*	*SE*	*t*	Sig.	*R*^2^
Image agreement	−149.13	30.1	−4.94	0.0001	0.31
AoA	89.63	17.830	5.03	0.0001	
Conceptual familiarity	−51.50	20.85	−2.47	0.014	
	**Name Agreement > = 0.50**				
Visual complexity	103.17	27.19	3.79	0.0001	0.37
Name agreement	−294.26	120.19	−2.45	0.016	
Image agreement	−89.0	43.23	−2.06	0.042	
AoA	43.741	21.24	2.060	0.042	

**Table 4:** Step-wise regression with ToT as a dependent variable.

Variables	*B*	*SE*	*T*	Sig.	*R*^2^
Image agreement	−0.91	0.16	−5.76	0.0001	0.31
Name agreement	−0.98	0.31	−3.11	0.002	
	**Name Agreement > = 0.50**				
Image agreement	−0.70	0.13	−5.55	0.0001	0.31
Visual complexity	0.26	0.08	3.27	0.002	

## Discussion

With the aim of developing a useful tool for professionals in the field of communication disorders and researchers of speech production, this study provides normative data for the 295 most frequent stimuli from the ARASAAC pictographic system. For each stimulus, the modal name, two measures of name agreement (name agreement and H scores), visual complexity and conceptual familiarity scores, image agreement scores, proportion of TOTs, and RTs in production tasks were collected. Reliability and validity of these scales were shown to be adequate.

Importantly, this study provides the modal name obtained from a name production task for each selected pictogram. In general, there is a low level of overlapping between the modal names obtained in this study and the labels provided in the ARASAAC database (36% concurrence). This result is important because it indicates that the names that have traditionally been linked to each picture in the ARASAAC database may not be the most suitable. One possible reason for these discrepancies could be that the ARASAAC names are based on subjective criteria and not on controlled normative studies. From this view, this study is in accordance with the increasing trend in speech therapy, psychology, and related fields toward the integration of the best available research in applied practice (evidence-based practice approach).

Furthermore, results from the current study showed that image agreement, age of acquisition, and conceptual familiarity predicted naming times. Thus, stimuli with higher values of image agreement, conceptual familiarity, and early acquisition were named more quickly. When only stimuli with higher levels of name agreement (higher than 0.50) were selected, visual complexity and name agreement also entered in to the model, indicating that those stimuli were less visually complex, produced higher levels of agreement in their names, and were more rapidly named. Regarding the ToT responses, results indicated that image agreement and name agreement modulated the number of ToTs (more image agreement and more name agreement produced fewer ToTs). When stimuli with higher levels of name agreement were selected (values higher than 0.50), image agreement and visual complexity entered in to the model, indicating that higher values of image agreement and less visual complexity elicited a reduced number of ToTs. These patterns of results in the regression analyses are relevant because they demonstrate that the set of indices collected in the current study are suitable modulators of speech processes. These data are also in accordance with principal models of speech production in that, with differences in the conceptualization of the specific mechanisms, they support the assumption that picture naming involves the retrieval of different types of information: the visual information extracted from the picture, the meaning of the depicted stimulus, and the lexical and phonological information associated with the intended target word in order to initiate the articulatory motor program of the name (Dell, 1986; Caramazza, 1997; Levelt et al., 1999). In this context, the ease of processing in production acts at the level of the structural stage of object recognition for the visual complexity and image agreement dimensions related to the uncertainty of pictures (Vitkovitch and Tyrrell, 1995; Bonin et al., 2002; Cheng et al., 2010). Meanwhile, conceptual familiarity is assumed to take place at the semantic/conceptual level (Perret and Bonin, 2018). At the subsequent level of lexical access, the name agreement related to the activation of more than one alternative name arises (Vitkovitch and Tyrrell, 1995; Bonin et al., 2002; Cheng et al., 2010) when the correct name needs to be selected from among competing alternatives (Kan and Thompson-Schill, 2004a,b; Shao et al., 2014). During the selection, nouns that are acquired early seem to have a more consolidated connection within the speech production system (Ellis and Lambon Ralph, 2000; Belke et al., 2005; Navarrete et al., 2013).

The current dataset provides valuables indices to be employed in different speech-related domains. Its validity, however, can be limited to the geographical and linguistic context where the norms are being collected. In this line, researchers of linguistic materials have opted to develop specific normative studies in different countries. For example, after the seminal work of Snodgrass and Vanderwart that provided normative data for a set of pictures in an English-speaking population, researchers from different countries developed new normative studies on these stimuli in Spanish (Sanfeliu and Fernandez, 1996; Cuetos et al., 1999; Moreno-Martínez and Montoro, 2012), in English (Barry et al., 1997), in French (Alario and Ferrand, 1999), in Mandarin Chinese (Liu et al., 2011) and in Russian (Tsaparina et al., 2011). Even in the case of Spanish-speaking countries other than Spain, the generalization of the current norms must be made with caution.

## Conclusion

This dataset is expected to facilitate communication processes for those people who demand visual support in their interaction with the environment, both in the field of disability (autism, cerebral palsy, cognitive deficit, aphasia, dementia, etc.), as well as in the social sphere (relations in hospital settings, retirement homes, or interlingual communication) since communication and language are essential for every human being and a fundamental right recognized by all international organizations (United Nations, 2006).

## Data Availability Statement

The raw data supporting the conclusions of this manuscript will be made available by the authors, without undue reservation, to any qualified researcher.

## Author Contributions

DP conceived the study. DP and AM designed the experiments, drafted the manuscript, and provided critical revisions. AM performed the statistical analysis. All authors approved the final version of the manuscript for submission.

## Conflict of Interest Statement

The authors declare that the research was conducted in the absence of any commercial or financial relationships that could be construed as a potential conflict of interest.

## Footnotes

1 http://www.arasaac.org/pictogramas_color.php

2 Notice that ToTs were not collected employing the standard procedure. In the standard ToT collection procedure, when a participant reports being in a ToT state, the target word appears in a recognition test to distinguish between positive and false ToTs (e.g., Gollan and Brown, 2006). However, in the current normative study, we did not have any *a priori* correct item to be used as a target in the recognition phase, since all possible responses were potentially correct. For example, if participants are presented by the picture called “human” (humano.png) in the ARASAAC database, different correct responses can be generated (body, man, human being, silhouette, etc). If the label “human” being is selected as a target, responses from participants that suffered ToTs when tried to recover other correct labels (body, silhouette, etc) would not be considered as positive ToTs, decreasing artificially the total number of ToTs experiences in the study. This limitation is important because there is a wide mismatch (64%) between the label assigned in the ARASAAC database and the label that we obtained after the written name agreement phase. Thus, we did not include this recognition phase in the ToT task procedure (see Marful et al., 2018, for a similar procedure), and the raw number of ToTs were included in the analysis. These data, in consequence, must be interpreted with caution (see Gollan and Brown, 2006, for a description of the possible limitations of this approach).

3 Name agreement was included in the analysis following the procedure of Bonin et al. (2008). Following the suggestion of an reviewer, we also included H (instead of name agreement) in the regression analysis. Results resembled the previously observed pattern with name agreement, and the same variables were included in the best models (with the exception that, when the previous model entered name agreement, H statistics were added) when testing RTs (image agreement, *B* = −149.13, *SD* = 30.17, *t*(189) = −4.94, *p* = 0.0001; AoA, *B* = 89.63, *SD* = 17.8, *t*(189) = 5.03, *p* = 0.0001, and conceptual familiarity, *B* = −51.50, *SD* = 20.85, *t*(189) = −2.47, *p* = 0.014, R^2^ = 0.31) and ToTs (image agreement, *B* = −0.97, *SD* = 0.15, *t*(192) = −6.35, *p* = 0.0001; H statistics, *B* = −0.22, *SD* = 0.08, *t*(192) = 2.65, *p* = 0.009, R^2^ = 0.30).

## Supplementary Material

TABLE S1 Normative data for 295 ARASAAC pictures.

## References

[B1] Alario F. X. Ferrand L. (1999). A set of 400 pictures standardized for French: norms for name agreement, image agreement, familiarity, visual complexity, image variability, and age of acquisition. *Behav. Res. Methods Instrum. Comput.* 31 531–552. 10.3758/BF03200732 10502875

[B2] Alario F.-X. Ferrand L. Laganaro M. New B. Frauenfelder U. H. Segui J. (2004). Predictors of picture naming speed. *Behav. Res. Methods* 36 140–155. 10.3758/BF03195559 15190709

[B3] Barry C. Morrison C. M. Ellis A. W. (1997). Naming the snodgrass and vanderwart pictures: effects of age of acquisition, frequency, and name agreement. *Q. J. Exp. Psychol. A* 50 560–585. 10.1080/783663595

[B4] Belke E. Brysbaert M. Meyer A. S. Ghyselinck M. (2005). Age of acquisition effects in picture naming: evidence for a lexical–semantic competition hypothesis. *Cognition* 96 B45–B54. 10.1016/j.cognition.2004.11.006 15925568

[B5] Beukelman D. Mirenda P. (2013). *Augmentative and Alternative communication: Supporting Children and Adults with Complex Communication Needs.* Baltimore, MA: Paul H. Brookes Publishing Co.

[B6] Bliss C. K. (1965). *Semantography: Blissymbolics.* Sydney, NSW: Sematography Publications.

[B7] Bonin P. Barry C. Méot A. Chalard M. (2004). The influence of age of acquisition in word reading and other tasks: a never ending story? *J. Mem. Lang.* 50 456–476. 10.1016/j.jml.2004.02.001

[B8] Bonin P. Chalard M. Méot A. Fayol M. (2002). The determinants of spoken and written picture naming latencies. *Br. J. Psychol.* 93 89–114. 10.1348/000712602162463 11839103

[B9] Bonin P. Peereman R. Malardier N. Méot A. Chalard M. (2003). A new set of 299 pictures for psycholinguistic studies: french norms for name agreement, image agreement, conceptual familiarity, visual complexity, image variability, age of acquisition, and naming latencies. *Behav. Res. Methods Instrum. Comput.* 35 158–167. 10.3758/BF03195507 12723790

[B10] Bonin P. Perret C. Méot A. Ferrand L. Mermillod M. (2008). Psycholinguistic norms and face naming times for photographs of celebrities in French. *Behav. Res. Methods* 40 137–146. 10.3758/BRM.40.1.137 18411536

[B11] Cabello F. Bertola E. (2015). Características formales y transparencia de los símbolos pictográficos de ARASAAC. *Rev. Inv. Logop.* 5 60–70.

[B12] Caramazza A. (1997). How many levels of processing are there in lexical access? *Cogn. Neuropsychol.* 14 177–208. 10.1080/026432997381664

[B13] Cheng X. Schafer G. Akyürek E. G. (2010). Name agreement in picture naming: an ERP study. *Int. J. Psychophysiol.* 76 130–141. 10.1016/j.ijpsycho.2010.03.003 20227446

[B14] Cuetos F. Alija M. (2003). Normative data and naming times for action pictures. *Behav. Res. Methods Instrum. Comput.* 35:168 10.3758/BF03195508 12723791

[B15] Cuetos F. Ellis A. W. Alvarez B. (1999). Naming times for the snodgrass and vanderwart pictures in Spanish. *Behav. Res. Methods Instrum. Comput.* 31 650–658. 10.3758/BF03200741 10633980

[B16] Cycowicz Y. Friedman D. Rothstein M. Snodgrass J. (1997). Picture naming by young children: norms for name agreement, familiarity, and visual complexity. *J. Exp. Child Psychol.* 65 171–237. 10.1006/jecp.1996.2356 9169209

[B17] Davies R. Barbón A. Cuetos F. (2013). Lexical and semantic age-of-acquisition effects on word naming in Spanish. *Mem. Cognit.* 41 297–311. 10.3758/s13421-012-0263-8 23180310

[B18] Dell G. S. (1986). A spreading-activation theory of retrieval in sentence production. *Psychol. Rev.* 93 283–321. 10.1037/0033-295X.93.3.283 3749399

[B19] Díez E. Fernandez A. Alonso M. A. (2014). *NIPE: Normas e ìndices de interès en Psicologìa Experimental.* Available at: http://campus.usal.es/gimc/nipe/

[B20] Duchon A. Perea M. Sebastiàn-Gallès N. Martì A. Carreiras M. (2013). EsPal: one-stop shopping for Spanish word properties. *Behav. Res. Methods* 45 1246–1258. 10.3758/s13428-013-0326-1 23468181

[B21] Ellis A. W. Lambon Ralph M. A. (2000). Age of acquisition effects in adult lexical processing reflect loss of plasticity in maturing systems: insights from connectionist networks. *J. Exp. Psychol. Learn. Mem. Cogn.* 26 1103–1123. 10.1037/0278-7393.26.5.1103 11009247

[B22] Ellis A. W. Morrison C. M. (1998). Real age-of-acquisition effects in lexical retrieval. *J. Exp. Psychol. Learn. Mem. Cogn.* 24 515–523. 10.1037//0278-7393.24.2.515 9530847

[B23] Fraga I. Padrón I. Perea M. Comesaña M. (2017). I saw this somewhere else: the Spanish Ambiguous Words (SAW) database. *Lingua* 185 1–10. 10.1016/j.lingua.2016.07.002

[B24] Glaser W. R. (1992). Picture naming. *Cognition* 42 61–105. 10.1016/0010-0277(92)90040-O 1582161

[B25] Gollan T. H. Brown A. S. (2006). From tip-of-the-tongue (TOT) data to theoretical implications in two steps: when more TOTs means better retrieval. *J. Exp. Psychol. Gen.* 135 462–483. 10.1037/0096-3445.135.3.462 16846276

[B26] Gómez-Veiga I. Carriedo N. Ruciàn M. Vila J. O. (2010). Estudio normativo de ambigüedad lèxica en castellano, en niños y en adultos (Norming study on lexical ambiguity in Spanish, in children and adults). *Psicologica* 31 25–47.

[B27] Guasch M. Boada R. Ferrè P. Sànchez-Casas R. (2013). NIM: a web-based Swiss army knife to select stimuli for psycholinguistic studies. *Behav. Res. Methods* 45 765–771. 10.3758/s13428-012-0296-8 23271155

[B28] Hartje W. Hannen P. Willmes K. (1986). Effects of visual complexity in tachistoscopic recognition of Kanji and Kana symbols by German participants. *Neuropsychologia* 24 297–300. 10.1016/0028-3932(86)90065-5 3714037

[B29] Hehner B. (1980). *Blissymbolics for Use.* Toronto, ON: Blissymbolics Communication Institute Pub.

[B30] Hirsh K. W. Funnell E. (1995). Those old, familiar things: age of acquisition, familiarity, and lexical access in progressive aphasia. *J. Neurolinguistics* 9 23–32. 10.1016/0911-6044(95)00003-8

[B31] Johnson R. (1981). *The Picture Communication System.* Solana Beach, CA: MayerJohnson Co.

[B32] Kan I. P. Thompson-Schill S. L. (2004a). Effect of name agreement on prefrontal activity during overt and covert picture naming. *Cogn. Affect. Behav. Neurosci.* 4 43–57. 10.3758/CABN.4.1.43 15259888

[B33] Kan I. P. Thompson-Schill S. L. (2004b). Selection from perceptual and conceptual representations. *Cogn. Affect. Behav. Neurosci.* 4 466–482. 10.3758/CABN.4.4.466 15849891

[B34] Lausen B. Krolak-Schwerdt S. Böhmer M. (eds) (2005). *Data Science, Learning by Latent Structures, and Knowledge Discovery.* Heidelberg: Springer.

[B35] Levelt W. J. Roelofs A. Meyer A. S. (1999). A theory of lexical access in speech production. *Behav. Brain Sci.* 22 1–38. 10.1017/S0140525X99001776 11301520

[B36] Liu Y. Hao M. Li P. Shu H. (2011). Timed picture naming norms for Mandarin Chinese. *PLoS One* 6:e16505. 10.1371/journal.pone.0016505 21298065 PMC3027682

[B37] Marful A. Díez E. Fernandez A. (2015). Normative data for the 56 categories of battig & montague (1969) in Spanish. *Behav. Res. Methods* 47 902–910. 10.3758/s13428-014-0513-8 25159692

[B38] Marful A. Dìez-Álamo A. M. Plaza-Navas S. Fernandez A. (2018). A normative study for photographs of celebrities in Spain. *PLoS One* 13:e0197554. 10.1371/journal.pone.0197554 29768497 PMC5955507

[B39] Moreno-Martínez F. J. Montoro P. R. (2012). An ecological alternative to snodgrass and Vanderwart: 360 high quality colour images with norms for seven psycholinguistic variables. *PLoS One* 7:e37527. 10.1371/journal.pone.0037527 22662166 PMC3360784

[B40] Navarrete E. Scaltritti M. Mulatti C. Peressotti F. (2013). Age-of-acquisition effects in delayed picture-naming tasks. *Psychon. B. Rev.* 20 148–153. 10.3758/s13423-012-0310-2 22893113

[B41] Palao S. (2013). *ARASAAC Symbol Dictionary. Portal aragonés de la comunicación aumentativa y alternative.* Available at: http://www.arasaac.org/

[B42] Perret C. Bonin P. (2018). Which variables should be controlled for to investigate picture naming in adults? A Bayesian meta-analysis. *Behav. Res. Methods.* 10.3758/s13428-018-1100-1 30066263

[B43] Romero Corral D. Marcos Rodrigo J. M. (2016). “Portal Aragonés de la Comunicación Aumentativa y Alternativa (ARASAAC),” in *Paper Presented at the Workshop Arasaac: Portal Aragoné*s de la Comunicación Aumentativa y Alternativa. Software, Herramientas y Materiales para la Comunicación, Otros recursos para CAA: Soyvisual. University of Granada.

[B44] Romski M. Sevcik R. A. Barton-Hulsey A. Whitmore A. S. (2015). Early intervention and AAC: what a difference 30 years makes. *Augment. Altern. Commun.* 31 181–202. 10.3109/07434618.2015.1064163 26153901

[B45] Sanfeliu M. C. Fernandez A. (1996). A set of 254 Snodgrass-Vanderwart pictures standardized for Spanish: norms for name agreement, image agreement, familiarity, and visual complexity. *Behav. Res. Methods Instrum. Comput.* 28 537–555. 10.3758/BF03200541

[B46] Schneider W. Eschman A. Zuccolotto A. (2012). *E-Prime User’s Guide.* Pittsburgh, PA: Psychology Software Tools, Inc.

[B47] Shao Z. Roelofs A. Acheson D. J. Meyer A. S. (2014). Electro- physiological evidence that inhibition supports lexical selection in picture naming. *Brain Res.* 1586 130–142. 10.1016/j.brainres.2014.07.009 25219485

[B48] Snodgrass J. G. Vanderwart M. (1980). A standardized set of 260 pictures: norms for name agreement, image agreement, familiarity, and visual complexity. *J. Exp. Psychol. Hum. Learn.* 6 174–215. 10.1037/0278-7393.6.2.174 7373248

[B49] Snodgrass J. G. Yuditsky T. (1996). Naming times for the Snodgrass and vanderwart pictures. *Behav. Res. Methods* 28 516–536. 10.3758/BF03200540

[B50] Tsaparina D. Bonin P. Méot A. (2011). Russian norms for name agreement, image agreement for the colorized version of the Snodgrass and Vanderwart pictures and age of acquisition, conceptual familiarity, and imageability scores for modal object names. *Behav. Res. Methods* 43 1085–1099. 10.3758/s13428-011-0121-9 21717267

[B51] United Nations (2006). *Convention on the Rights of Persons with Disabilities.* Available at: https://www.un.org/development/desa/disabilities/convention-on-the-rights-of-persons-with-disabilities.html 10.1515/9783110208856.203 18348362

[B52] Vitkovitch M. Tyrrell L. (1995). Sources of disagreement in object naming. *Q. J. Exp. Psychol.* 48A, 822–848. 10.1080/14640749508401419

[B53] Yoon C. Feinberg F. Hu P. Gutchess A. H. Hedden T. Chen H.-Y. M. (2004). Category norms as a function of culture and age: comparisons of item responses to 105 categories by American and Chinese adults. *Psychol. Aging* 19 379–393. 10.1037/0882-7974.19.3.379 15382989

